# First clinical experience in man with the IMRICOR-MR-EP system:Electrophysiology study guided by real-time MRI

**DOI:** 10.1186/1532-429X-14-S1-P205

**Published:** 2012-02-01

**Authors:** Matthias Gutberlet, Matthias Grothoff, Charlotte Eitel, Christian Lücke, Philip Sommer, Christopher Piorkowski, Gerhard Hindricks

**Affiliations:** 1 Diagnostic and Interventional Radiology, University Leipzig - Heart Centre, Leipzig, Germany; 2 Cardiology -Rhythmology, University Leipzig - Heart Centre, Leipzig, Germany

## Background

Magnetic resonance imaging (MRI) in the context of electrophysiology (EP) studies facilitates visualization of complex three-dimensional anatomy with the respective underlying arrhythmia substrate, real-time visualization of functional informations and complications, as well as lesion visualization during ablation under elimination of radiation exposure. In the following we present our first experience of a real-time MRI guided EP study demonstrating current possibilites and drawbacks.

## Methods

Five patients (4 male, 1 female; mean age 64.4 years (±10.2)) with symptomatic arrhythmias, 3 with highly symptomatic typical atrial flutter, presented to our hospital for isthmus ablation, 1 for an electrophysiology study (EP) and 1 for slow pathway ablation in AV-NRT. The four ablations were performed successfully and complete bidirectional isthmus block was confirmed in 3 patients with atrial flutter. After the conventional procedure in the EP lab all five patients were transferred to a 1.5 T whole body MRI scanner (Intera, Philips, Best, The Netherlands) for an EP diagnostic procedure. Two MRI compatible steerable diagnostic/ablation catheters (VisionTM, Imricor Medical Systems, Burnsville, MN, USA) were inserted via the femoral sheaths and manipulated by an experienced electrophysiologist. Using a commercially available interactive real-time steady-state free precession MRI sequence (TR=3 ms, TE=1ms, flip angle=35°, slice thickness=10mm, frame rate=8 per second).

## Results

**Figure 1:**
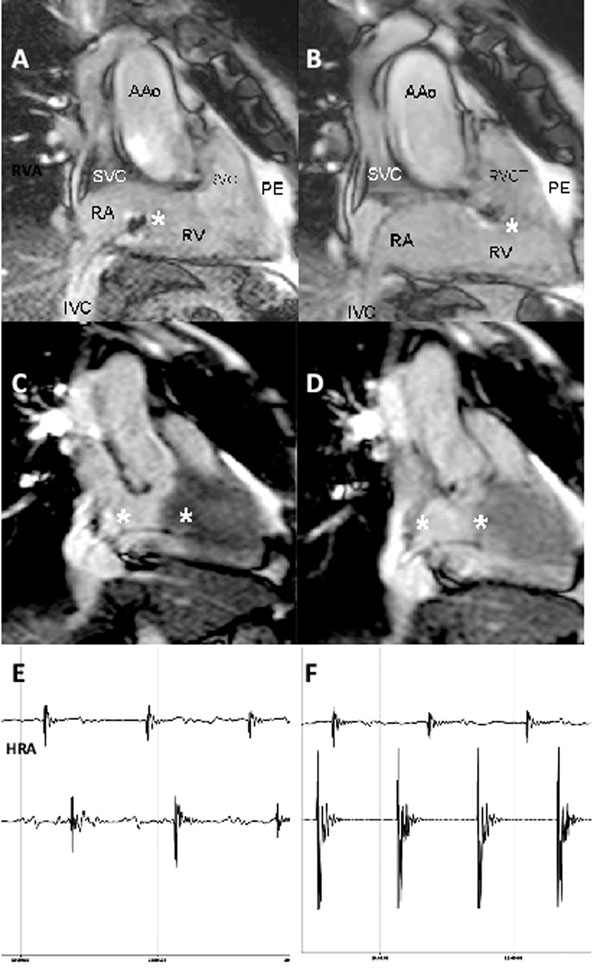


Using passive catheter tracking all catheters could be placed successfully in the right ventricle (Figure 1. Panel A, B; RAO view) and in the right atrium (Panel C, D; RAO view) confirmed by intracardiac electrograms (Panel E). Furthermore, simple programmed stimulation maneuvers were performed (Panel F, ventricular pacing). During and after the procedure no adverse effects were observed in all patients.

## Conclusions

To our knowledge this is the first pilot study of real-time MRI guided placement of multiple catheters in humans with subsequent performance of stimulation maneuvers. Besides the mentioned benefits this technology still encounters several limitations, which have to be solved before application in a routine clinical setting. Challenges arise from delineation of precise surface ECG recordings in the MRI setting along with intracardiac electrograms, easier handling of catheters, visualization of catheters placed in the coronary sinus, facilitation of immediate defibrillation in the MRI setting and implementation of an active catheter tracking system. Developments are under way moving this promising technology closer.

## Funding

The equipment was provided by the IMRICOR company.

